# Efficacy of Short‐Term Timed Artificial Insemination Protocols With Estradiol Esters and PGF2α in Girolando Cows

**DOI:** 10.1111/rda.70076

**Published:** 2025-05-15

**Authors:** Mariana dos Santos Dutra Okada, Lara Nogueira Silenciato, Samuel Rodrigues Bonamichi do Couto, Joaquim Esquerdo Ferreira, Marco Roberto Bourg de Mello

**Affiliations:** ^1^ Universidade Federal Rural do Rio de Janeiro Seropédica Rio de Janeiro Brazil; ^2^ Centro Universitário de Barra Mansa Barra Mansa Rio de Janeiro Brazil; ^3^ UNIFAA Centro Universitário de Valença Valença Rio de Janeiro Brazil

**Keywords:** conception rate, dairy cattle, ovulation rate, short‐term ovulation synchronisation

## Abstract

Using a synchronisation protocol based on estradiol esters and prostaglandin, established in cattle since the 1970s, we implemented 24 or 48‐h intervals between hormone administration and timed artificial insemination (TAI). This study compared two short‐duration TAI protocols using estradiol benzoate (EB) and cypionate (EC) as ovulation inducers. A total of 172 Girolando female cows were selected after ovarian ultrasonographic evaluation and received 500 μg of prostaglandin F2α (PGF) at the start of the treatment (D0). The inclusion criterion for females in the study was the presence of a corpus luteum measuring ≥ 14 mm and at least one follicle with a diameter between 8 and 20 mm. The cows were randomly sorted into two treatment groups. The EB group received 2 mg of EB 24 h after PGF (D1), and TAI was performed 24 h later (D2). The EC group received 2 mg estradiol cypionate simultaneously with PGF, and TAI was performed 48 h later. Pregnancy was diagnosed 30 days after AI using transrectal ultrasonography. Experiment I evaluated follicular dynamics in 18 females (EB: *n* = 9; EC: *n* = 9) by analysing the follicular growth rate, pre‐ovulatory follicle (POF) diameter, ovulation timing and ovulation rate. No statistically significant differences were observed (*p* > 0.05), with a 77.8% ovulation rate in both treatments. Experiment II compared the conception rates in 172 females (EB: *n* = 85; EC: *n* = 87). The overall conception rate was 30.2%, with no significant difference between the treatments (EB: 27.1%; EC: 33.3%). However, cows with a body condition score < 3.0 tended to have higher conception rates with EC than with EB (39.1% vs. 18.2%; *p* = 0.06). Cows with POF ≥ 10 mm at D0 had significantly higher conception rates with EC than with EB (48.3% vs. 29.0%; *p* = 0.03). Both protocols were equally effective; however, EC was more advantageous for cows with a POF ≥ 10 mm at treatment initiation.

## 1 Introduction

Crossbreeding between Holstein and Gir cattle has been employed as an effective strategy to enhance milk production and reproductive performance in pasture‐based systems under tropical conditions. The thermotolerance of Zebu cattle is primarily attributed to their lower basal metabolic rate and greater heat dissipation capacity, facilitated by increased peripheral vasodilation, sweating rate and respiratory frequency. Currently, approximately 80% of the Brazilian dairy herd consists of Holstein–Gir crosses, collectively referred to as the Girolando breed, a composite breed developed through selective crossbreeding (Mendonça et al. 2025).

Timed artificial insemination (TAI) is a reproductive technique widely used in dairy farming to improve herd reproductive efficiency. However, the success of TAI can be influenced by several factors, such as body condition score, female cyclicity at the start of the protocol, quality of hormonal protocols, herd health and inseminator experience and commitment (Mercadante and Lamb 2024).

Numerous TAI protocols are available for cattle herds in Brazil, and the selection of the most appropriate strategy depends on factors such as breed, animal category, reproductive and nutritional status, as well as physiological variables, including the interval from the LH surge to ovulation, the specific hormonal regimen used, and other underlying physiological conditions. Traditional protocols are often extended in duration (10–12 days) and involve multiple hormone applications and handling events. Therefore, research aimed at simplifying these protocols, reducing the interval between the initiation of hormonal treatment and insemination, and minimising management interventions is of considerable value. Such improvements may facilitate broader adoption of the technique and enhance reproductive efficiency by optimising the timing of insemination within the ideal breeding window.

In this context, short‐term ovulation synchronisation protocols have been developed since the 1970s, particularly following the studies conducted by Roche (1977), with the aim of reducing costs and labour requirements. These protocols typically include the administration of prostaglandin F2α (PGF) and estradiol during the initial phase of the treatment regimen. These protocols are most effective in female cattle that possess a functional corpus luteum (CL), as prostaglandin F2α (PGF) is the most effective luteolytic agent for inducing luteolysis in the presence of an active CL (McArt et al. 2010). Therefore, the administration of agents capable of synchronising the emergence of a new follicular wave—such as gonadotropin‐releasing hormone (GnRH) or estradiol benzoate—approximately 7 days prior to PGF treatment enhances the luteolytic efficiency of PGF by ensuring the presence of a responsive CL at the time of administration.

In early studies using short‐duration ovulation synchronisation protocols, a combination of different hormones was tested as an ovulation inducer, all of which were administered after PGF treatment to induce an LH surge, and consequently, ovulation (Roche 1977; Stevenson et al. 2003; Cirit et al. 2008; Jeong et al. 2013; McArt et al. 2010). López‐Gatius (2000) reported a pregnancy rate of 39% in postpartum dairy cows subjected to AI 48 h after PGF treatment, combined with 250 IU of human chorionic gonadotropin and 1 mg of estradiol benzoate (EB) 12 h after PGF treatment. Bandai et al. (2020) evaluated ovulation synchronisation using short‐duration protocols in lactating dairy cows, utilising EB and gonadotropin‐releasing hormone (GnRH) to minimise costs and labour in the TAI protocol. In this study, a TAI protocol for dairy cattle was developed, involving hormone administration and TAI at a 48 h interval with only two management interventions, aiming for an efficiency comparable to that of the three‐handling short protocol.

EB and estradiol cypionate (EC) differ in pharmacokinetics, with EB exhibiting rapid absorption and a short half‐life, resulting in a brief estrogenic effect, whereas EC, due to its more lipophilic structure, is absorbed more slowly and has a prolonged half‐life, leading to sustained oestrogen release (Vynckier et al. 1990). This extended hormonal stimulation with EC likely supports the final maturation and timely ovulation of larger dominant follicles present at treatment initiation, enhancing synchrony with insemination. Therefore, initial follicular size is a key factor in selecting the appropriate ovulation inducer, with EC conferring a reproductive advantage in cows with physiologically advanced follicles.

The objective of this study was to compare the reproductive efficiency of a short‐duration TAI protocol for ovulation synchronisation in dairy cattle using EB or estradiol cypionate (EC) as ovulation inducers. This study tested the following hypotheses: (i) there is no difference in ovulation and conception rates between short‐duration TAI protocols using EB or EC as ovulation inducers and (ii) cows with a pre‐ovulatory follicle (POF) ≥ 10 mm at the start of treatment have significantly higher conception rates when treated with EC than with EB.

## 2 Materials and Methods

The Animal Experiments Local Ethics Committee of the Universidade Federal Rural do Rio de Janeiro (UFRRJ) approved the animal experimental protocol (number: 0172‐07‐2022).

### 2.1 Animals and Ultrasound Evaluation

A total of 172 crossbred cows (Holstein–Gir), with an average body condition score (BCS) of 3.0 ± 0.0, from three commercial herds located in the southeastern region of Brazil, were evaluated between January 2022 and June 2023. The study was conducted on farms where artificial insemination was exclusively performed by trained and experienced technicians. Cows were milked twice daily, with an average annual milk yield of 4270 kg per cow during the experimental period. The animals were managed under a semi‐intensive system, characterised by pasture‐based feeding supplemented with either a total mixed ration or a combination of forage and grains during periods of feed scarcity. Fresh water was provided ad libitum. The animals were selected by gynaecological examinations with the aid of an ultrasound device (SonoScape, DM10V, linear transducer; 7.5 MHz), via the transrectal route. To select the females for inclusion in this study, ultrasound evaluation of the ovaries was performed in B mode, in which the CL diameters and the largest FL (dominant or POF) present at the time of evaluation were measured. The diameters were calculated as the average of the two largest transverse measurements (perpendicular to each other) performed on each structure. To be included, the female had to have a CL diameter ≥ 14 mm and at least one FL with a diameter of 8–20 mm (adapted from Bandai et al. 2020).

### 2.2 Short‐Term Protocols for Ovulation Synchronisation and Pregnancy Diagnosis

**FIGURE 1:**
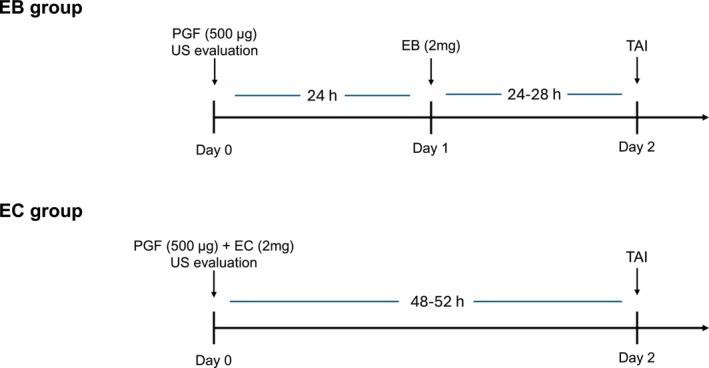
Hormonal protocol used in animals belonging to the estradiol benzoate (EB) and estradiol cypionate (EC) groups. CL, corpus luteum; PGF, prostaglandin F2α; TAI, timed artificial insemination; US, ultrasound.

All animals that met the inclusion criteria (corpus luteum ≥ 14 mm and follicle diameter between 8 and 20 mm) received an intramuscular injection of 500 μg of cloprostenol (2.0 mL of Sincrocio, Ourofino Saúde Animal, Cravinhos, SP, Brazil) on Day 0 (D0) of the protocol. After PGF application, the females were randomly distributed into two treatment groups: EB and EC. Females in the EB Group received (im) 2 mg of EB (2.0 mL of Sincrodiol, Ourofino Saúde Animal, Cravinhos, SP, Brazil) 24 h after cloprostenol application, and after 24 h, TAI was performed. Females in the EC Group received (im) 2 mg of EC (2.0 mL of SincroCP, Ourofino Saúde Animal, Cravinhos, SP, Brazil) simultaneously with the application of cloprostenol, and after 48 h, TAI was performed (Figure 1). The pregnancy diagnosis was performed 30 days after AI, using transrectal ultrasonography (SonoScape, DM10V, linear transducer; 7.5 MHz). Pregnancy confirmation was based on the visualisation of the embryonic vesicle in the presence of a viable embryo (presence of heartbeat).

### 2.3 Experimental Design

This study was conducted as two trials (Experiments I and II). In Experiment I, the follicular dynamics of 18 females subjected to short‐term protocols for ovulation synchronisation were evaluated (Groups EB and EC both had nine animals). From the beginning of each hormonal treatment, the animals were subjected to ultrasound evaluations (SonoScape, DM10V, linear transducer; 7.5 MHz), always performed transrectally by the same operator, every 12 h, to determine the follicular growth rate (mm/day), POF diameter, time and rate of ovulation. In each ultrasound evaluation, the positions of the ovarian structures (CL and follicles) and their diameters were recorded on cards for subsequent analyses. Ovulation was considered (confirmed) when the POF disappeared between two consecutive ultrasound examinations. The following parameters were evaluated: follicular growth rate (mm/day); POF diameter (mm), considering the last measurement taken at the time of insemination; time of ovulation in relation to PGF application as well as in relation to TAI (hours); and ovulation rate (%). In Experiment II, 172 females were subjected to two synchronisation protocols (Groups EB and EC with 85 and 87 animals, respectively) to compare conception rates, that is, the number of pregnancies divided by the number of cows subjected to each protocol (P/TAI). The semen used for insemination procedures was sourced from eight different bulls.

### 2.4 Statistical Analysis

For the statistical analysis, EB and EC treatments, cyclicity, body condition score, category, farm, inseminator, bull and interactions were included in the model. For the final model, the variables were removed based on the Wald statistical criterion when *p* > 0.20. All continuous variables were tested for normality using the Shapiro–Wilk test. Response variables, such as the interval between removal of the P4 device and ovulation (hours), interval between TAI and ovulation (hours), diameter of the dominant follicle (DF) on D0 and D2 and DF growth rate (mm/day), were compared using ANOVA through the Biostat 5.3 program. The proportions of synchronised, ovulated, and pregnant cows according to treatment were analysed using the nonparametric binomial test in the BioEstat 5.3 software. The interactions between conception rates, BCS, and the diameters of CL and POF were evaluated by categorising cows based on these reproductive parameters at the start of the synchronisation protocol (D0). Specifically, conception rates were compared among cows divided into four groups according to combinations of CL (≥ 20 mm or < 20 mm) and POF (≥ 10 mm or < 10 mm) diameters. This test assesses differences between two proportions from independent samples to determine whether the observed difference (*p*_1_ – *p*_2_) is sufficiently large to reject the null hypothesis of equal proportions. The size of each sample was sufficient for the normal curve to approximate the binomial distribution; that is, the two conditions were met: *n*1*p*1 *q*1 ≥ 5 and *n*2*p*2 *q*2 ≥ 5. Significant differences were considered for *p* < 0.05 (5% significance), while differences between *p* > 0.05 and *p* < 0.10 were considered as a trend. Data are presented as means ± SDs and percentages for continuous and binary outcomes, respectively.

## 3 Results

The results regarding the follicular dynamics of females subjected to the two short protocols using EB or EC are presented in Table 1. No significant differences (*p* > 0.05) were observed for any of the evaluated parameters.

**TABLE 1:** Mean parameters (±SD) related to the follicular dynamics of crossbred dairy cows (Girolando) submitted to two short ovulation synchronisation protocols, using estradiol benzoate or cypionate as ovulation inducers.

Parameters	Estradiol benzoate group (*n* = 9)	Estradiol cypionate group (*n* = 9)	*p*
PGF—OV (h)	69.4 ± 9.1	72.8 ± 18.1	0.66
TAI—OV (h)	21.4 ± 9.1	25.7 ± 16.5	0.56
DFGR (mm/dia)	0.9 ± 0.3	0.6 ± 0.5	0.11
FLD0 (mm)	9.8 ± 1.8	10.6 ± 1.2	0.28
FLD2 (mm)	11.3 ± 2.2	11.7 ± 1.9	0.66
OR (%)	77.8 (7/9)	77.8 (7/9)	1.00
BCS	3.2 ± 0.1	2.9 ± 0.1	

Abbreviations: BCS, body condition score; DFGR, dominant follicle growth rate; FLD0, dominant follicle diameter on Day 0; FLD2, dominant follicle diameter on day TAI; OR, ovulation rate; OV, ovulation; PGF, prostaglandin; TAI, timed artificial insemination.

The conception rate, follicle diameter at the beginning of the protocol, and CL on D0 (Experiment II) are presented in Table 2. No significant differences (*p* > 0.05) were observed between treatments (EB and EC) for any of the parameters evaluated.

**TABLE 2:** Mean diameters (±SD) of the dominant follicle and corpus luteum measured at the beginning of the protocol and mean conception rate of crossbred dairy cows (Girolando) subjected to two short ovulation synchronisation protocols.

Parameters	Estradiol benzoate group	Estradiol cypionate group	*p*
FLD0 (mm)	11.5 ± 2.2	11.0 ± 1.8	0.16
CLD0 (mm)	19.3 ± 3.0	19.6 ± 3.5	0.51
CR (%)	27.1 (23/85)	33.3 (29/87)	0.37
BCS	3.0 ± 0.0	3.0 ± 0.0	

Abbreviations: BCS, body condition score; CLD0, diameter of the corpus luteum at the time of PGF application; CR, conception rate; FLD0, dominant follicle diameter on Day 0.

Table 3 compares the conception rates between the two treatments (EB and EC) according to the factors that could interfere with the protocol success. The conception rates were compared considering the body condition score (BCS ≥ or < 3.0), the diameter of the dominant follicle (POF ≥ or < 10 mm) and the corpus luteum (CL ≥ or < 20 mm) measured at the time of PGF application and at the time of AI. Additionally, the conception rates of cows separated into four combinations between the CL and POF diameters were compared (CL ≥ 20 mm and POF ≥ 10 mm; CL < 20 mm and POF < 10 mm; CL ≥ 20 mm and POF < 10 mm; CL < 20 mm and POF ≥ 10 mm). A significant difference (*p* = 0.03) in conception rates was observed between the two treatments only when follicle size (≥ 10 mm) on D0 was considered. Considering only females with follicles > 10 mm at the beginning of synchronisation (D0), the conception rate was significantly higher in females in the EC group compared to the EB group (48.3% and 29.0%, respectively). Additionally, cows in the EC group tended (*p* = 0.06) to have higher conception rates when BCS < 3 or a combination of CL diameter with POF at the beginning of the protocol (CL ≥ 20 and POF ≥ 10 mm) were considered. In contrast, the CL diameter at the beginning of hormonal treatment (D0) had no impact on the conception rate. Similarly, the bull and location did not interfere with the conception rate in either treatment group. When comparing conception rates within EB or EC according to the factors previously mentioned (BCS, CL and POF diameters on D0), no statistical difference was observed for any factor within the EB group, that is, conception rates were the same regardless. Conversely, within the EC group, there was a tendency for a higher conception rate in cows that presented follicles ≥ 10 mm (*p* = 0.06) or CL ≥ 20 mm (*p* = 0.07) in diameter at the beginning of the synchronisation treatment.

**TABLE 3:** Conception rates of crossbred dairy cows (Girolando) subjected to two short ovulation synchronisation protocols according to body condition score (BCS) and corpus luteum (CL) diameters and preovulatory follicle (POF) measured at the beginning of the treatment.

Parameters	EB group conception rate (%)	EC group conception rate (%)	*p*
BCS ≥ 3	30.1 (19/63)	31.2 (20/64)	0.89
BCS < 3	18.2 (4/22)	39.1 (9/23)	0.06
*p*	0.27	0.49	
CL ≥ 20 mm	25.0 (8/32)	40.0 (14/35)	0.09
CL < 20 mm	28.3 (15/53)	28.8 (15/52)	0.95
*p*	0.73	0.27	
POF ≥ 10 mm	29.0 (18/62)	48.3 (28/58)	0.03
POF < 10 mm	21.7 (5/23)	27.6 (8/29)	0.62
*p*	0.5	0.06	
CL ≥ 20 mm and POF ≥ 10 mm	20.0 (5/25)	40.0 (10/25)	0.06
CL < 20 mm and POF < 10 mm	12.5 (2/16)	20.0 (4/20)	0.5
CL ≥ 20 mm and POF < 10 mm	42.8 (3/7)	40.0 (4/10)	0.9
CL < 20 mm and POF ≥ 10 mm	35.1 (13/37)	33.3 (11/33)	0.43
*p*	0.09	0.07	

## 4 Discussion

To the best of our knowledge, no previous study has evaluated the efficiency of short‐term protocols for ovulation synchronisation in Girolando cattle using EB and EC as ovulation‐inducing agents. Although the absence of a control group using follicular wave synchronisation could be viewed as a limitation of this study, the results nonetheless demonstrate the feasibility of implementing abbreviated ovulation synchronisation protocols in dairy cows. These findings contribute to the simplification and acceleration of timed artificial insemination (TAI) programmes. Notably, one of the evaluated protocols (EC group) required only two handling events, highlighting its potential for practical application in commercial dairy settings.

Here, the intervals between prostaglandin application and ovulation, or even between TAI and ovulation, did not differ between the EB and EC treatments, a finding in agreement with that reported by Silva et al. (2019), where similar times were also observed between the removal of the P4 device and ovulation using the two ovulation inducers (EB and EC). These researchers also used Girolando cows; however, the duration of the ovulation synchronisation protocol was 10–11 days (traditional ovulation synchronisation protocol). The results of the present study, as well as those of Silva et al. (2019), show that EC applied at the time of device removal and benzoate—used 24 h after device removal—had the same effect. This difference in hours of application occurs due to the shorter half‐life of benzoate, which induces the availability of 17β‐estradiol more quickly. The fact that there was no significant difference in the timing of ovulation between the two inducers used in the short synchronisation protocols in dairy cows allowed for a reduction of 1 day of management in this short protocol, enabling the performance of TAI with only two treatments (D0 = PGF + EC; D2 = TAI).

Regarding the follicular growth rate, the absence of a significant difference between cows treated with EB and EC (0.95 ± 0.31 vs. 0.62 ± 0.51 mm/day, respectively) is consistent with the findings of Silva et al. (2019) in Girolando cows, although that study used long‐term protocols (11 days). Follicular growth rate in cattle is modulated by several factors that influence the development and diameter of the POF, such as nutritional status, breed, animal category and seasonal conditions.

In Experiment I, the diameter of the POF on the day of TAI was 11.26 ± 2.22 mm for the EB group and 11.71 ± 1.95 mm for the EC group, with no difference observed between them. This was also observed in a study by Franca et al. (2015), which was also conducted with crossbred Holstein × Gir lactating females, and no statistical difference was observed between the follicle diameters measured on the day of insemination for the EB and EC groups (respectively, 11.45 ± 2.34 mm and 10.71 ± 2.43 mm). The similarity between the POF diameters in the EB and EC groups in the two studies was expected because the size of the follicle at the time of ovulation is considerably influenced by genetics.

The similar ovulation rates observed in the groups treated with EB and EC in the present study differ from results reported by Silva et al. (2019), in which EB was more effective than EC as an ovulation inducer in Girolando females (90.4% and 71.4%, respectively). According to these authors, this difference was attributed to the greater capacity of EB to increase 17‐β‐estradiol concentrations in the bloodstream earlier on, thus favouring higher ovulation rates. The discrepancy between the findings of the two studies can be explained by the difference in the duration of the protocols employed and the use of intravaginal progesterone devices in the study by Silva et al. (2019), as the presence of progesterone influences follicular development and, consequently, the ovulation rate. It is also important to note that the present study was not specifically powered to detect statistical differences in ovulation rate, and we recognise this as a limitation when interpreting these results.

The conception rate of dairy cows subjected to TAI is influenced by several factors including body condition score, milk production level, herd health status, reproductive management, semen quality and workforce training. In general, conception rates in dairy cows range from 30% to 50% (Almeida et al. 2016; Stevenson et al. 2006), with higher values achieved when good reproductive management practices are adopted. Previous studies have corroborated this variation. Bandai et al. (2020) reported conception rates between 29.8%–56.6% in dairy cows synchronised with short‐duration protocols (52–76 h) using GnRH or EB as ovulation inducers. Similarly, López‐Gatius (2000) compared three short protocols using human chorionic gonadotropin and EB as ovulation inducers in dairy cows and observed conception rates ranging from 31% to 39%. Therefore, although the conception rates obtained here could be improved, they remained within the expected efficiency range for this reproductive biotechnique in dairy cattle.

A potential strategy for improving conception rates in dairy cows is the use of hormones that promote the growth of the dominant follicle to a larger diameter at the time of TAI. One example is equine chorionic gonadotropin, which acts directly during the final phase of follicular development. An increase in the diameter of the dominant follicle during synchronisation protocols can result in higher ovulation and conception rates because follicles with larger diameters tend to form larger and more functionally competent corpora lutea with a greater capacity for progesterone (P4) production, which favours a uterine environment more conducive to embryo implantation. Another viable approach would be the administration of GnRH at the time of TAI in females that present low estrus expression and smaller diameter dominant follicles at the end of the synchronisation protocol. This strategy can improve fertility under specific conditions, as demonstrated by Fernandes et al. (2024).

Here, no significant difference in the conception rate was observed between the two treatments. However, Bandai et al. (2020) compared the use of EB and GnRH as ovulation inducers in short TAI protocols in dairy cattle and found significant differences in conception rates (56.6% for EB and 29.8% for GnRH). The authors suggested that this difference could be attributed to the beneficial effects of estradiol at high concentrations during the pre‐ovulatory period. This positive effect of estradiol on fertility is in line with findings from other studies, which indicate that exposure to estradiol during the pre‐ovulatory period is essential for embryonic growth and placental attachment, as demonstrated in beef cows by Madsen et al. (2015). Here, both sources of estradiol (benzoate and cypionate) were used, which may have promoted similar hormone concentrations in the pre‐ovulatory period, thus justifying the absence of differences in conception rates between the treatments.

BCS is one of the most studied factors related to the success of TAI in dairy cattle. Here, when comparing conception rates in cows with BCS < 3, a trend of higher pregnancy rates was observed in the group treated with EC than in the group treated with EB. This trend can be explained by the greater availability of EC in relation to EB, considering its prolonged half‐life and its impact on the timing of ovulation (Dransfield et al. 1998; Silva et al. 2019). EC remains in the bloodstream of treated animals for longer periods, which may favour the synchronisation of ovulation and consequently improve conception rates.

When conception rates were compared considering some factors that could affect the efficiency of TAI, it was observed that in animals that had follicles ≥ 10 mm on D0, the conception rate was significantly higher in the EC group (48.3% vs. 29.0%). However, Silva et al. (2019) reported similar conception rates in Girolando cows when EB was compared with EC. Here, a possible explanation for the higher conception rate for the EC group—when considering only animals that had a POF ≥ 10 mm on D0—may be the greater availability of this hormone in the body due to its longer half‐life (Taghizadeh et al. 2024) and also that ovulation occurs at more favourable times for conception (Dransfield et al. 1998; Silva et al. 2019). These findings should be interpreted with caution due to the limited sample size within the subgroups. Further research involving larger and more stratified populations is necessary to determine the physiological relevance of these potential interactions.

When comparing the pregnancy rate in the group treated with EC between cows with POF ≥ 10 mm and those with POF < 10 mm on D0, a trend toward a higher conception rate was observed in females with POF ≥ 10 mm compared to those with smaller follicles. This trend can be explained by the fact that follicles with larger diameters tend to form larger corpora lutea, which have a greater capacity for progesterone production and create a more favourable uterine environment for maintaining pregnancy. In addition, cows with larger follicles have a greater probability of ovulation (Binelli et al. 2001), which contributes to an increased conception rate.

When comparing conception rates based on combinations of CL and POF diameters, a trend toward a higher conception rate was observed in the EC‐treated group (40%) compared to that in the EB‐treated group (20%), when cows had CL ≥ 20 mm and POF ≥ 10 mm on D0. This trend may be related to the longer half‐life of EC, which may promote ovulation at times more favourable for the conception period (Dransfield et al. 1998; Silva et al. 2019). Similar results were reported by Bandai et al. (2020), who observed differences in conception rates when comparing short synchronisation protocols, with a higher conception rate in the EB group than in the GnRH group. The authors attributed these results to the critical role of estradiol in the pre‐ovulatory period, which is essential for sustaining embryonic development and/or placental attachment in cattle. Furthermore, they reported that the use of EC in the pre‐ovulatory period may increase pregnancy rates in cows with dominant follicles of a smaller diameter.

Here, when comparing conception rates within the EC group, a trend of higher pregnancy rates was observed in females with CL ≥ 20 mm at the beginning of the protocol compared to those with CL < 20 mm. This trend can be attributed to prior exposure to higher levels of progesterone, which exerts two main effects: (1) reduced expression of oestrogen receptors in the hypothalamus, decreasing negative feedback on GnRH production and release, and (2) stimulation of oestrogen receptor expression in hypothalamic regions more sensitive to the effect of estradiol on luteinizing hormone secretion (Gümen and Wiltbank 2002; Simões et al. 2018). This mechanism occurs due to changes in both the quantity and location of oestrogen receptors in the hypothalamus, particularly in the mediobasal region (Blache et al. 1994). In addition, there is a known relationship between CL size and progesterone production in cattle, which may modulate LH release, thereby contributing to increased conception rates.

In short, TAI protocols for Girolando cows showed no difference in the conception rate compared to these inducers when the cows had functional corpora lutea on D0, indicating that these simple short‐term ovulation synchronisation protocols can be an interesting tool to enable quick and cheap TAI application in dairy cows. However, considering the difference in management between the two protocols, EC still has the advantage of having only two management procedures, which reduce labour, the likelihood of errors, or the chances of forgetting to administer the hormones, in addition to less management, which decreases stress to the animals. In general, this protocol optimises labour and time for professionals, in addition to increasing the chances of the female becoming pregnant within the desired service period, as the protocol time is reduced by an average of 7 days and also significantly reduces the cost.

## 5 Conclusions

Although there are pharmacological differences between the two ovulation‐inducing agents examined (EB and EC), both proved to be effective in synchronising ovulation, leading to comparable and satisfactory conception rates in dairy cattle using a short, practical and cost‐effective synchronisation protocol. Furthermore, if a follicle ≥ 10 mm is present at the start of the protocol, the use of EC is recommended.

This study provides valuable insights into optimising short‐duration TAI protocols by demonstrating that EC and EB yield similar overall conception rates, with EC offering advantages for cows with specific reproductive conditions. These findings contribute to improving reproductive management strategies in Girolando cattle, potentially enhancing fertility outcomes in targeted subgroups.

## Author Contributions

**Mariana dos Santos Dutra Okada:** writing – review and editing, writing – original draft, validation, investigation, formal analysis. **Lara Nogueira Silenciato:** writing – review and editing, validation, investigation, formal analysis. **Samuel Rodrigues Bonamichi do Couto:** writing – review and editing, resources, formal analysis. **Joaquim Esquerdo Ferreira:** validation, supervision. **Marco Roberto Bourg de Mello:** validation, supervision, project administration, investigation, funding acquisition, conceptualization.

## Conflicts of Interest

The authors declare no conflicts of interest.

## Data Availability

The data that support the findings of this study are available from the corresponding author upon reasonable request.
